# Calibration-Aimed Comparison of Image-Cytometry- and Flow-Cytometry-Based Approaches of Ploidy Analysis

**DOI:** 10.3390/s22186952

**Published:** 2022-09-14

**Authors:** Viktor Zoltán Jónás, Róbert Paulik, Miklós Kozlovszky, Béla Molnár

**Affiliations:** 1 Image Analysis Department, 3DHISTECH Ltd., 1141 Budapest, Hungary; 2 Department of BioTech Research Center, Óbuda University, 1034 Budapest, Hungary

**Keywords:** digital pathology, cytology, cytometry, calibration, ploidy analysis, DNA content

## Abstract

Ploidy analysis is the fundamental method of measuring DNA content. For decades, the principal way of conducting ploidy analysis was through flow cytometry. A flow cytometer is a specialized tool for analyzing cells in a solution. This is convenient in laboratory environments, but prohibits measurement reproducibility and the complete detachment of sample preparation from data acquisition and analysis, which seems to have become paramount with the constant decrease in the number of pathologists per capita all over the globe. As more open computer-aided systems emerge in medicine, the demand for overcoming these shortcomings, and opening access to even more (and more flexible) options, has also emerged. Image-based analysis systems can provide an alternative to these types of workloads, placing the abovementioned problems in a different light. Flow cytometry data can be used as a reference for calibrating an image-based system. This article aims to show an approach to constructing an image-based solution for ploidy analysis, take measurements for a basic comparison of the data produced by the two methods, and produce a workflow with the ultimate goal of calibrating the image-based system.

## 1. Introduction

Analyzing a cell population based on its DNA content is an everyday task for cytology labs today. The aim is ploidy analysis, which provides information about the populations of entities in the sample based on their DNA content. This information is fundamental in the diagnosis of cancer. Although DNA sequencing technologies have emerged and developed since its existence, DNA ploidy analysis remains a dominant method, as it provides answers to diagnostically relevant questions in a simple and quick manner. Flow cytometry (FCM), described in [1], and more vividly illustrated—albeit on plant material—in [2], analyses a sample in a fluid form, making the long-term storage of the original input data (i.e., the cell population itself) non-viable. Image-based technologies (e.g., image cytometry (ICM)), described in detail in [3], have the benefit in terms of storage that they obtain more information in the acquisition phase, opening the possibility of reanalyzing the sample later. One approach is to modify flow cytometers for imaging (IFC), as described in [4], although the storage aspect here is still not addressed usually; another approach is to use motorized microscopes and/or glass slide scanners for cytometry, which is the method used in this paper. Comparison of the results of the two methods for different use cases for validation is performed frequently [5], showing similar [6] and very similar results to this work [7]. This opens the possibility of not only reproducing the exact same results, but also using new techniques for extracting more information from the images for the reassessment of the samples.

The acquisition phase and the exact methodology we employed to extract the DNA content information from the propidium iodide (PI)-stained samples through image analysis were described previously in [8,9,10,11], and a validation study was also conducted to compare this approach to results achieved manually by experts in terms of concordance with the cell-level image segmentation level [12].

The next step towards the goal of a well-founded ICM-based ploidy analysis is to construct a method for calibrating the ICM system; this article aims to inspect possibilities regarding this direction.

## 2. Materials and Methods

### 2.1. Samples Analyzed

The assay was based on 17 specimens of healthy human blood containing only PI-stained cell nuclei. These were digitized using a glass slide scanner produced by 3DHISTECH Ltd., Budapest, Hungary (Pannoramic Scan, fluorescent setup, 5MP sCMOS camera, 40* (Carl Zeiss AG, Jena, Germany) objective, and an LED-based lighting method to excite the fluorochrome). The resulting resolution of the samples was 0.1625 µm per pixel (jpeg compressed). Three of the twenty scanned digital slides from the specimens were not sufficient in quality (these samples were used to tune the digitization method and parameters, and were not comparable to the other samples by the end of the process due to dye bleaching). These three samples were excluded from the assay. The FCM measurements were conducted using a Becton Dickinson FACSCAN flow cytometer with the CellQuest software (version 3.1, both hardware and software by Becton Dickinson, Franklin Lakes, NJ, USA). The samples were stained with PI for visualization of DNA content.

The ICM measurements were taken on subsamples of these samples, sampled as demanded by lab protocols, in amount that would fit on a glass-covered glass slide. The samples were identified by their sequential indices throughout the assay. Care was taken to avoid any changes to sample quality during preparation, handling, and storage in the time interval between the two measurements.

### 2.2. Analysis Method

To achieve calibration, three straightforward methods were considered:

The first was to conduct cell-by-cell measurements with both FCM and ICM approaches, and form mathematical relations based on the results. This method seems to be non-viable because of the nature of the measurements. To achieve this, each object would need to be uniquely identifiable in both approaches’ results to be able to pair and compare the two approaches, and this is not feasible within the technological limits of these measurements, mainly because both are constructed to conduct a statistical type of analysis with a great number of entities, and no simple (and, thus, financially viable) method is available to label these entities.

Another method would be to artificially create calibrating samples from multiple types of plastic beads of appropriate size and fluorescence properties, in a way that would be useful in the calibration process. The linearity of intensity with regard to changes in exposure was analyzed in [13,14]; we used fluorescent beads to analyze the intensity relationship between the ICM (scanning fluorescence microscopy (SFM) in the referred article) and FCM methods. The drawbacks of this approach are its price and out-of-workflow nature. The calibration must be conducted at regular intervals, while temporarily stopping the everyday laboratory workload.

The third possible method is to analyze the results of both methods (ICM and FCM) for a real sample with known properties, composed of multiple populations of objects, and find a method to define a mathematical relation between the two, thus creating a transfer function that describes the relation between the two approaches. This article aims to show findings regarding this third approach.

In this investigation, we chose the healthy sample to be the real-world sample with known properties as the calibration data. Healthy samples are a usual portion of the data analyzed every day and, thus, are a good candidate for this purpose. Of course, these are biological samples; as such, they are heterogeneous by nature [15]. Their special property is containing two populations of entities that generate two peaks on the DNA content histogram. These, in turn, have a special relationship, where one peak represents double the amount of DNA of the other peak.

In the case of the selected method, there is one more level of complexity: the FCM samples are too great in volume to be placed on the glass slides analyzed by the ICM method. This means that the ICM method used subsamples of them, which were digitized via a digital microscope, thus creating the input data used in this article. The act of taking a blood sample from the patient is for now considered to be analogous to the creation of the subsample of the FCM sample for the ICM analysis (regular laboratory practices were used to prepare the glass slides with the subsamples, where dilution and division of fluid samples are everyday tasks—consider a patient’s blood sample going through multiple types of analyses).

The segmentation, as presented in previous works, was implemented as a plugin for the application QuantCenter (3DHISTECH Ltd., Budapest, Hungary).

For data manipulation and statistical evaluation, MATLAB (version R2019b, MathWorks Inc., Natick, MA, USA) was used.

The FCM generated one data file per sample for all of the samples. The ICM results were also extracted into one data file per sample. 

The pairs of ICM–FCM measurements were analyzed in parallel. The FCM results were kept unaltered and used as the reference.

#### 2.2.1. Flow Cytometry Output

A regular flow cytometry analysis contains a few measurement channels. There are usually two detectors, which provide a forward scatter (FSC) and a side scatter (SSC) measurement. Each detector provides the time function of the voltage of the detector for the passing of a measured object between the light source and the detector. These functions are described with measurements such as maximum, width, and area (integral). The FSC area is proportional to the object size, while SSC is proportional to the internal complexity (i.e., granularity) of a cell. Multiple light sources (of different spectra) can be used, and the resulting light (in case of using special fluorescent staining) may be filtered again through band-pass filters. In our case, the FSC area measurement was enough to capture the ploidy histogram generated by the appropriate lighting and filtering for the PI staining.

#### 2.2.2. Image Cytometry Output—DNA Content

**Figure 1:**
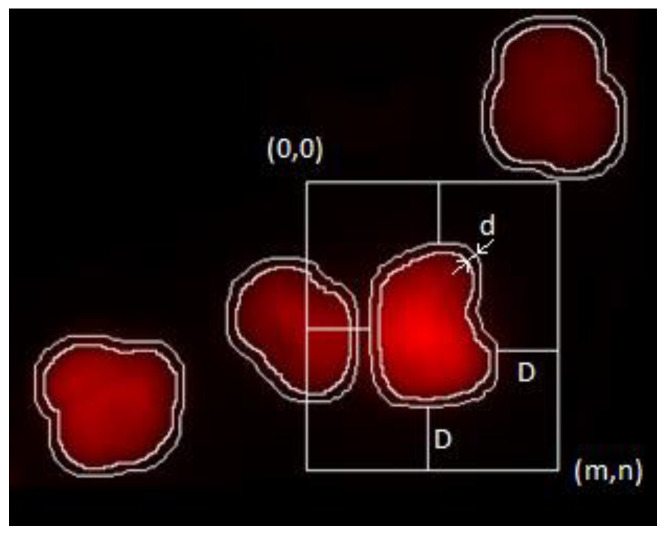
Method of background intensity measurement: the resolution of the system is visible; the objects are only tens of pixels in size.

The ploidy analysis itself consists of the analysis of the DNA content histogram of the measured population. The staining used is designed to produce intensity proportional to the amount of DNA present at a given location. As the observed intensity is proportional to the DNA content, the DNA content of the objects measured (in our case, the cell nuclei [16]) is defined as the integrated intensity of the nuclei, normalized against the background intensity as displayed on Figure 1.

This measurement was conducted on a grayscale digital image, and in discrete format, as follows:

(1) $$ID=\sum_{i=j=1}^{i=m,j=n}((I_{m,n}*J_{m,n})-\bar{BkgInt})$$

where *ID* is the integrated density, and *I* denotes the image intensities in the *m,n* neighborhood.

If *O_i_* is the currently analyzed object:

(2) $$O_{i}=\cup J_{m,n}I_{m,n}$$

then *J* is an indicator function that has the value of 1 when it is a pixel corresponding to the measured object *O_i_*, and 0 otherwise. 

*BkgInt* designates the background intensity, which was approximated by the average non-object intensity around each object (sample and image tile-level background approximation was examined and rejected based on visual inspection; dye accumulation regions and changes in background intensity was observed). The following is a simple average of intensities:

(3) $$\bar{BkgInt}=\frac{\sum B}{\left|B\right|}$$

where *B* is the set of background pixel intensities.

(4) $$\sum B=\sum_{i=j=1}^{i=m,j=n}K_{m,n}L_{m,n}I_{m,n}$$

Here, *L’* indicates the complement set of all non-foreground pixels:

(5) $$L^{\prime }=\overset{\left|O\right|}{\underset{i=0}{\cup }}O_{i}$$

*K* is again an indicator function:

(6) $$K=\{\begin{array}{ll} 1, & if\;\bar{O_{i}B}>d\\ 0, & otherwise \end{array}$$

where the shortest distance between the current object *O* and background *B* is greater than parameter *d*.

The neighborhood *m,n* was defined to ensure at least *D* distance from *O_i_*.

*D* was selected to be 35 pixels (the average object size in pixels at this magnification) so as to exclude other entire objects from the neighborhood; *d* was selected to be 5 pixels based on visual inspection (this is the distance where bulk of the effect of object proximity is eliminated from intensity values).

### 2.3. Image Cytometry Output—Object Area

The measurement of the area of an object using image-processing-based segmentation can be performed simply through counting the number of pixels that constitute the object. In this case, labeled masks were used to designate the different objects and the background region. If real-world size is important, it can be multiplied by the scale of the processed image. The FCM method uses a relative scale (the integral of the curve generated by the object passing in front of the detector).

### 2.4. Image Cytometry Output—Object Granularity

In the ICM approach, only one channel was recorded, but the location information retained in the planar images enabled the construction of a model of these measurements. Because there is no means to trace every measurement point of the FCM-measured solution to the image location of the ICM approach, the heuristics described below were used. The sample preparation caused the DNA to unwind from chromosomes to the well-known double-helix form, to be able to stain the DNA in such a way that the visible stain was proportional to the amount of the DNA. This caused the cell nuclei to show on the ICM images as cohesive, separated, well-formed objects. This enabled the usage of the area of these objects as the model of the FSC measurement of the FCM approach (both being a measure of the size/area of the object). 

The SSC is a measure of the inner granularity of the objects. Through image processing to capture this property, a summarized gradient type of measurement seemed appropriate to model the original SSC measurement. Discrete morphological gradient [17] and edge detection methods—Roberts, Prewitt, Sobel, and Canny operators [18] (p. 728)—were inspected. The evaluation was based on clustering (k-means). The goal was to find the best model for SSC that enabled the most robust clustering of the populations in the area–SSC model space throughout the 17 samples. Cluster gap values [19,20] were measured using MATLAB to find the best cluster count for all samples. A scoring system was used to determine the best candidate; this consisted of a simple fault count—a difference from the majority vote among all of the edge detection algorithms’ results was considered a fault. The algorithm with the least faults was selected.

## 3. Results

### 3.1. DNA Content

**Figure 2:**
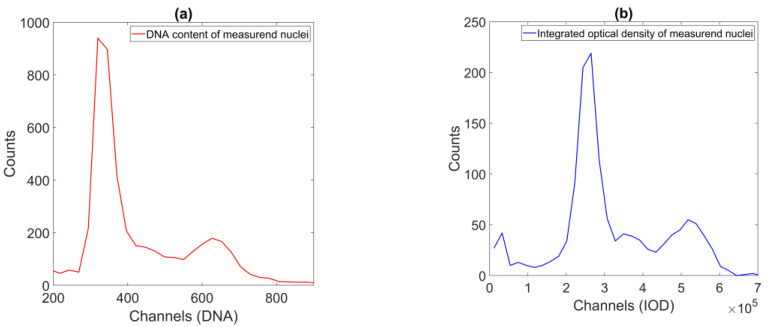
FCM (**a**) and ICM DNA content (**b**) histograms of one of the samples. Both *x*-axis values are intensity sums: in the FCM case, the maximal integral recorded at the detector during a nucleus passthrough; in the ICM case, the sum of the pixel intensities belonging to the segmented nucleus region (corrected by approximated background intensity). The dual peaks typical of samples from healthy donors are identifiable on the plots of both methods.

The FCM data show the signature features of a (healthy patient’s) DNA sample—the two peaks, located roughly at one and two units from the origin on the horizontal axis. A similar shape is visible on the ICM-generated data, but with much more noise, and a less prominent second peak (Figure 2.).

**Figure 3:**
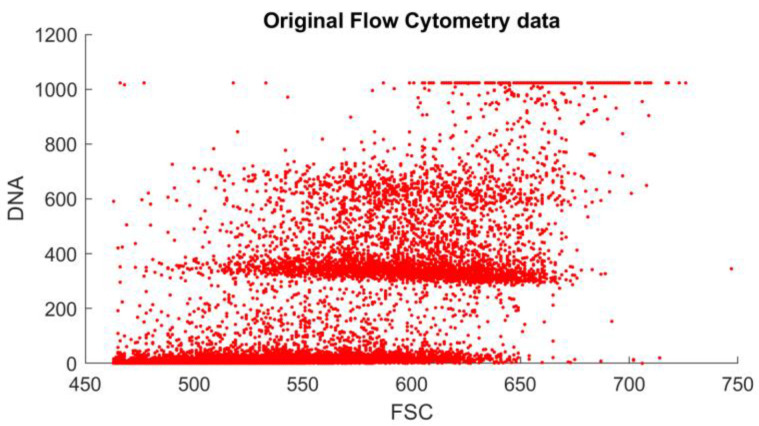
Flow cytometry data on a DNA content–object area scatterplot. Each data point represents a cell nucleus. The units of both axes are intensity-based: on the *y*-axis is the DNA content; on the *x*-axis is the forward scatter (a value proportional to the object size). The two populations of the 2N and 4N peaks are identifiable from looking at the *y*-axis.

**Figure 4:**
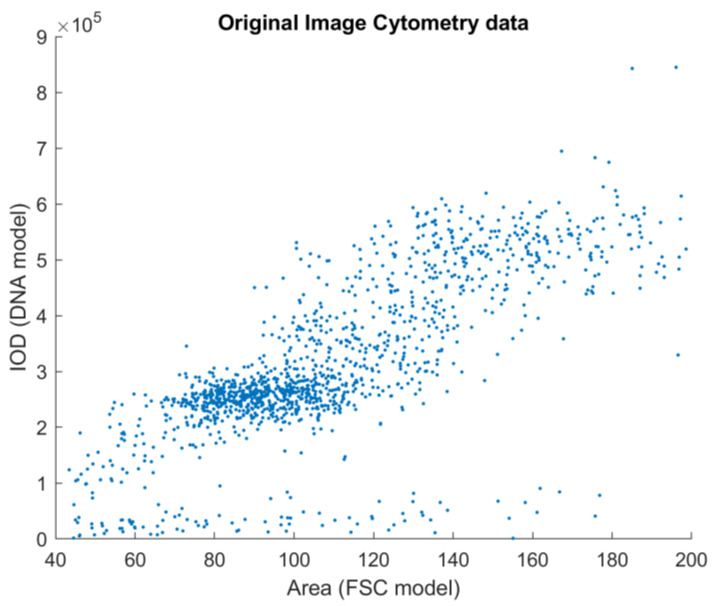
Image cytometry data on a DNA content–object area scatterplot. Each data point represents a cell nucleus. The unit of both axes are intensity-based: on the *y*-axis is the integrated optical density—the proposed model for the DNA content; on the *x*-axis is the object area, measured after segmenting the nuclei from the image. Some subpopulations are identifiable, but not as clearly as on the FCM plot.

Looking at these histograms for all 17 samples, similarities were visible. The ICM method uses only a single channel to generate these measurements, although it is two-dimensional. The ICM-measured DNA content of a nucleus is closely related to the segmentation results through the area of the object itself. The question emerged as to whether the two measurements were independent of one another. First, the scatterplots of these parameter pairs were visually inspected; samples are shown in Figure 3 and Figure 4.

To further investigate this suspicion, principal component analysis (PCA) [21] was carried out to measure this connection. To be able to compare all three cellular parameters of the FCM approach, an ICM measure for cell granularity was introduced.

### 3.2. Principal Component Analysis (PCA)

**Figure 5:**
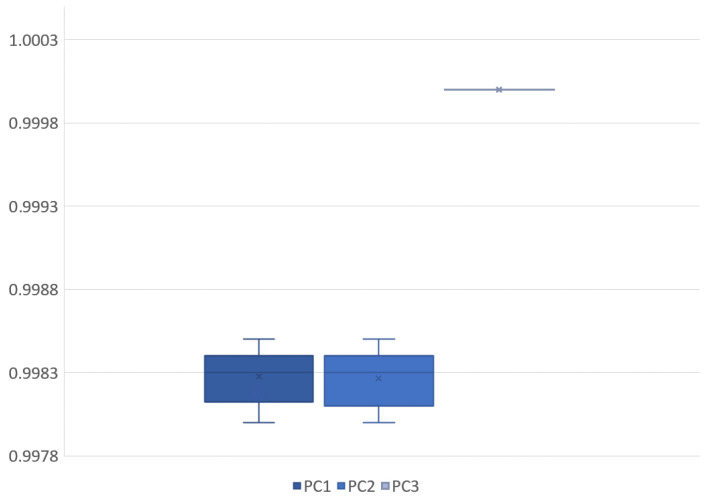
Principal component analysis (PCA) coefficient values across all 17 samples: The three input dimensions are area, granularity, and integrated fluorescence. Such high PCA coefficients mean that there is low dependence between these measured values, and they represent the associated measurements well. PC1 explains DNA content, PC2 explains granularity, and PC3 explains object area.

**Figure 6:**
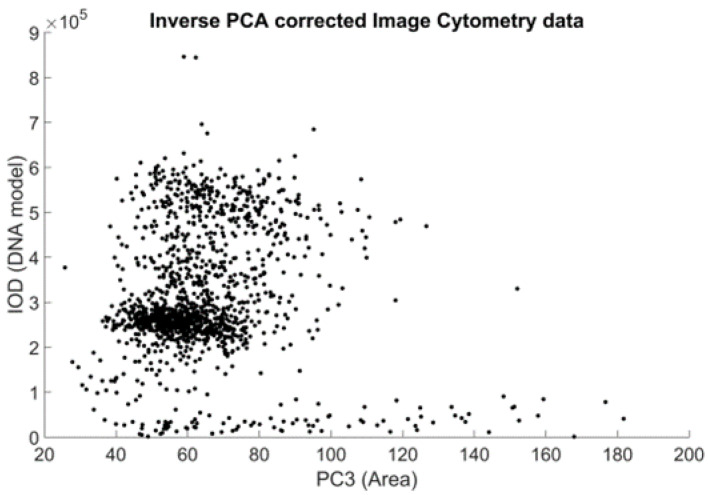
ICM data after the correction defined by the PCA: The resemblance to the FCM data plot (Figure 3) was improved. Each data point represents a cell nucleus, but now in the PC1–PC3 coefficient plane (representing the underlying, noise-filtered area, and DNA content measurements). The subpopulations of the 2N and 4N peaks are more prominent.

Based on the PCA analysis, the three proposed measurements seem to be close to their respective major principal components. Table 1 can be interpreted as positive feedback for the assumption that these proposed measurements are lightly correlated at most, with each adequately describing the purpose for which it was constructed. Figure 5 contains the visual summary of the principal components for all 17 samples. The PCA components define a transformation to model data better, the result of the correction of a sample is visible on Figure 6.

### 3.3. Granularity 

**Figure 7:**
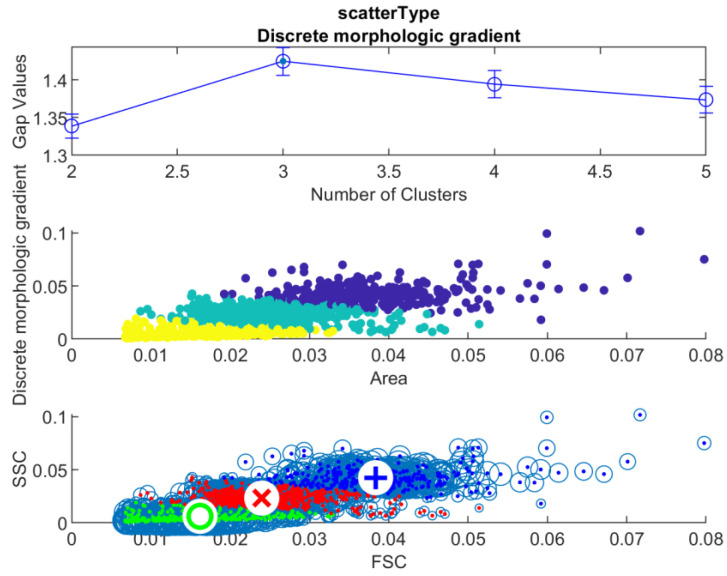
For each of the 17 samples, clustering gap analysis was conducted to approximate the possible object population counts. The results of a single sample are presented here with gap values (**top**), k-means method, with the different colors designating different populations (**middle**), and c-Means with the different colors designating different populations, with the cluster centers (**bottom**). These measurements were used to identify a model for the side scatter measurement of the FCM method that was proportional to the object granularity, with the internal structures identifiable.

A parameter called side scatter (SSC) is usually measured in the FCM method. This value is proportional to the measured object’s internal structure, its granular content. Figure 7 shows an overview of a selection method of a model parameter for this measurement for the ICM approach. Table 2 summarizes the inspected algorithms proposed as a measure of nuclear granularity.

### 3.4. Identification of Populations

**Figure 8:**
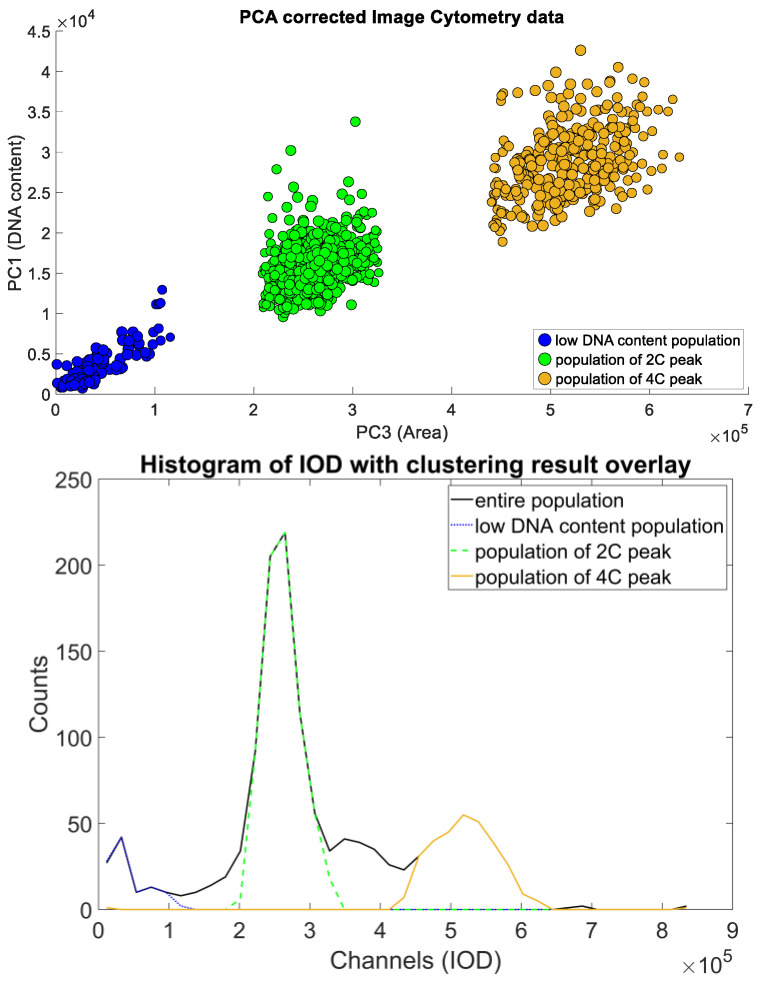
The results of c-means clustering on a single sample, with the clusters projected back to the DNA content distribution of the sample (blue, green, orange). These measurements were used to identify the approximate locations of the subpopulations of the 2N and 4N peaks.

The initial idea behind the construction is based on the identification of the two populations containing one and two units of DNA. These populations can be simply identified on the respective DNA content histograms of both the ICM and the FCM methods. Here, we used the PCA-filtered data already at hand to reduce the (already small) effect of the inherent correlation of the original measurements (Figure 8).

### 3.5. Curve Fitting to Cell Populations

**Figure 9:**
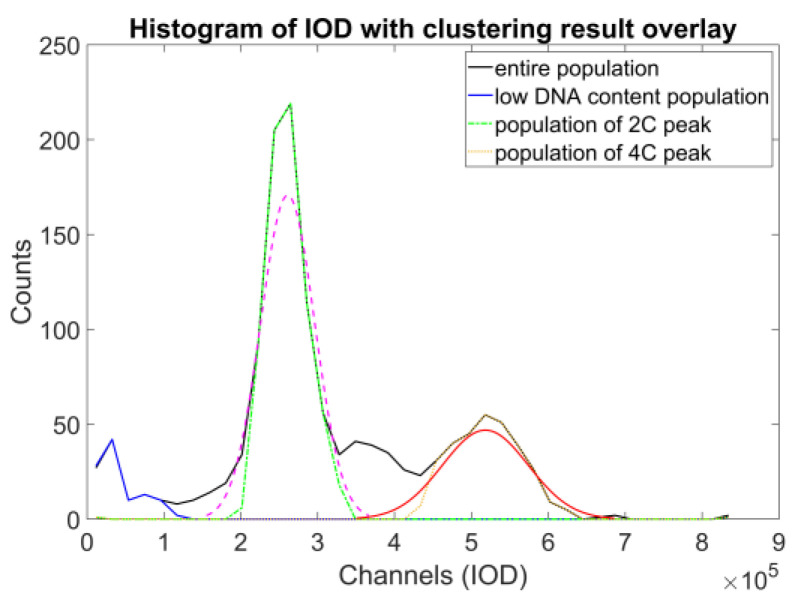
The DNA content distribution of sample #2, with the clusters projected back to the DNA content distribution of the sample (blue, green, orange), and normal distributions fitted to the latter two populations (magenta, red).

**Figure 10:**
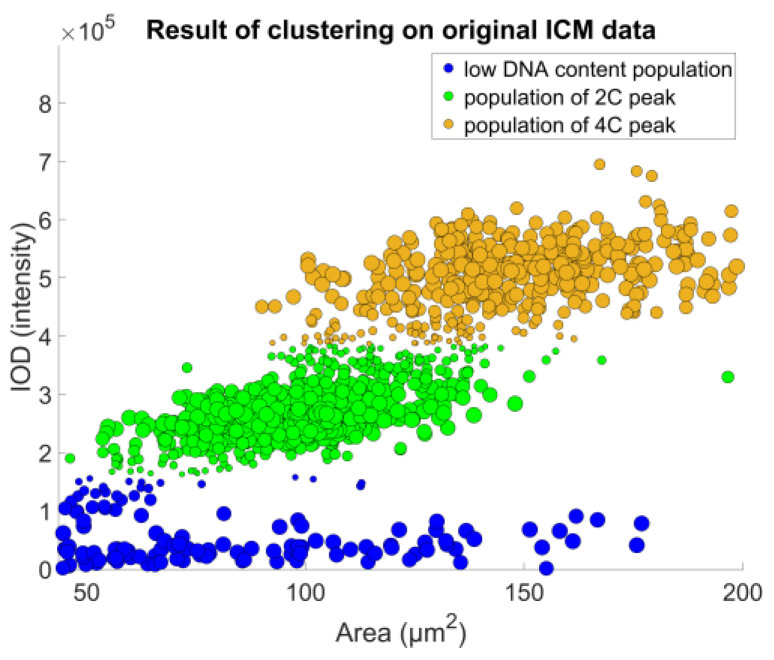
The original ICM data scatterplot recolored based on the clustering results: Data point radii represent the cluster membership value. Classification results identified the peak populations, and provided the basis of the measurement of their DNA content values.

The populations thus identified needed to be represented by their expected value (Figure 9) shows the curve fitting result of a single sample; projecting back clustering results on the original IOD-Area scatterplot for the same sample is visible on (Figure 10).

### 3.6. Calibration Research Results

**Figure 11:**
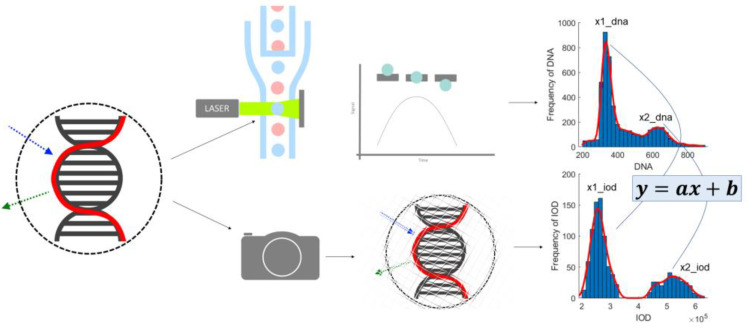
Overview of the reference (FCM) and the proposed (ICM) methods: The two approaches attempt to measure the same physical property through two different appliances. The ICM method was shown to have a linear response to uniform-intensity sources (i.e., fluorescent beads) with the parameter of exposure time (within the detection range of the appliance). Measuring nuclei, the same method is proposed to also have a linear relationship with the values produced by the reference method.

**Figure 12:**
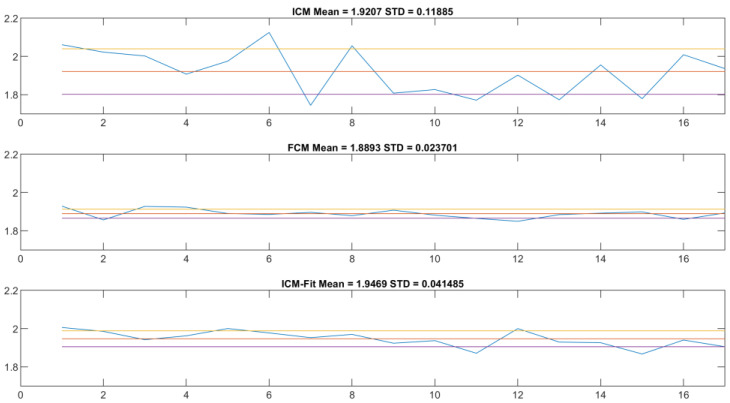
The ratio of the 2N and the 4N peak DNA content ratios for the histogram bin-based image cytometry (**top**), for flow cytometry (**middle**), and the reference and normal distribution fit-based image cytometry (**bottom**) results: The horizontal axis contains the 17 samples by index; the vertical axis shows the peak ratio value (the actual data with blue, the average with red, yellow and purple lines represent the ± 1 SD interval). The diagrams show the same range for comparison. The lower standard deviation of the bottom plot compared to the original (**top**) one is prominent.

**Figure 13:**
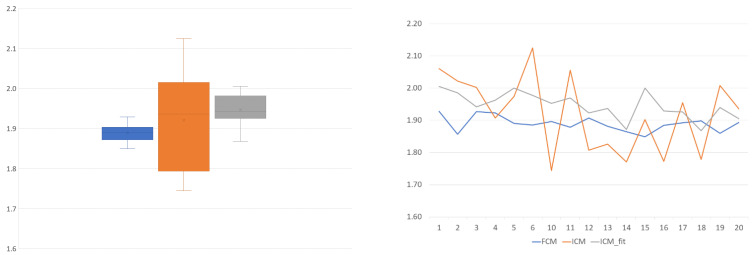
Graphical comparison of the peak ratios for the 17 samples in two equivalent representations: The datasets are marked with the same color on both plots. Peak ratio is the ratio of the DNA content at the 4N peak to the DNA content of the 2N peak. This theoretically equals 2.0 because, during mitosis, a normal body cell first replicates/doubles its DNA content before dividing into two new daughter cells.

**Figure 14:**
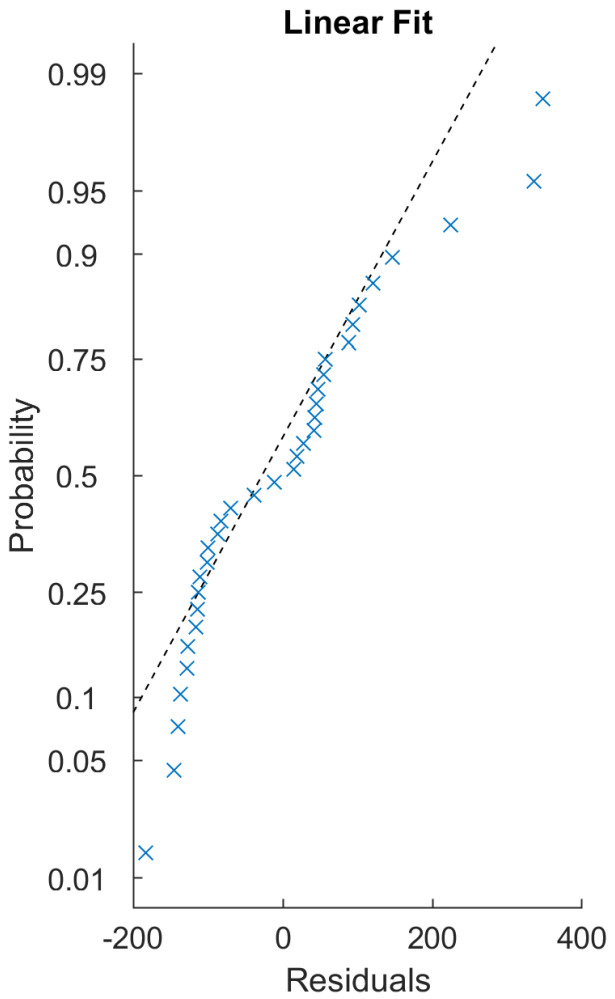
Graphical results of the linear regression for the more exact, ICM-fit dataset with the standard regression model: The data do not seem to align well with linear regression. Residuals seem to be non-normal. Removing hand-selected outliers based on overall image intensity did not improve the dataset considerably in this regard.

**Figure 15:**
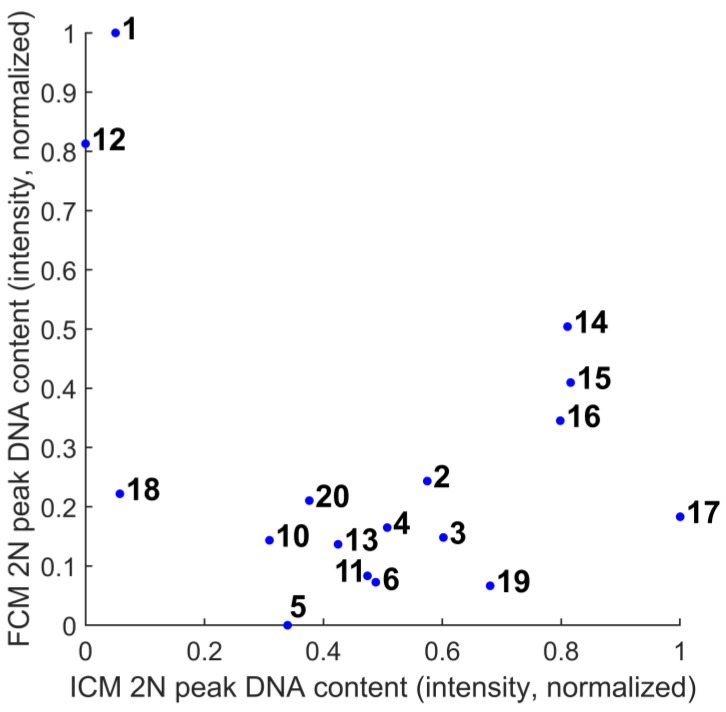
The DNA content of the first peaks of the samples (as a very rough approximation of the image intensity), the data points labeled with the sample indices. Samples #1, #12, and #18 are of lower intensity than the other samples. This is probably due to extended exposure to excitation light during multiple attempts to select the digitization parameters. These samples were treated as outliers for the rest of the process. Samples #7, #8, and #9 were not digitized in the expected quality (unfocused), and were omitted from the study altogether.

Figure 11 is a representation of the process of calibration. The next step is comparing the ICM and FCM methods from the point of view of these peak values statistically (Figure 12) and comparatively (Figure 13). The ultimate goal of calibration is to attempt to fit a model (Figure 14) to the transformation of the ICM peak points to the FCM data scale. Figure 15 is a visual help for the investigation of why fitting the model was unsuccessful.

## 4. Discussion

When creating an alternative method for an already-established process, it is mandatory to set milestones and compare the results of the proposed method to the reference. Between these milestones, a possible route is to model the reference process and validate it at the milestones. In the case of ploidy analysis and flow cytometry, the direct comparison is only possible at a few points in the process, and is statistical in nature. In [22], the authors successfully conducted a study specifically on esophageal cancer, in a similar population-based manner. In the first stage of this endeavor, the goal was to detect the objects—i.e., the cell nuclei—for later measurement. The second goal was to find a way to measure similar model parameters that approximate the reference measurements well, while the third was to compare the results achieved and create a way of defining the relationship between the reference and the proposed method. This article deals with the second and third goals.

The parameters of the reference method were reconstructed using the model parameters; these were provided by well-known image processing algorithms (Figure 1 and Figure 2). The median match ratio used for the granularity measure selection was based on the majority vote model.

The results generated by the reference and the proposed method can be compared visually (Figure 3 and Figure 4). Their similarity is visible, but the differences motivated the application of principal component analysis to identify and remove internal dependencies between the model parameters. The results showed that the model parameters were strongly independent of one another, and there seemed to be no unexplained variables present (see Table 1 and Figure 8). Applying the small noise filtering provided by PCA in this case, the similarity to the FCM result is slightly more prominent (Figure 6).

The denoised data were then used to identify the object populations defining the peaks of the DNA histogram. As the DNA content is a biochemical measure that contains noise, we decided to opt for a direct classification method that enables a robust approach—fuzzy c-means clustering, described in [23], and further detailed in [24] (pp. 133–149); the actual implementation was based on filtering the data points through classification uncertainty (Figure 8).

Using these populations, we could already compare the proposed method to the reference method. At first, we approximated the expected value of the DNA content of an object of a single population, using the bin center value of the DNA histogram’s highest corresponding peak. We compared the ratios of the two expected values of the two relevant populations (where the expected values were in 1:2 relation) to the FCM results (Figure 10, top and middle). These are the main anchor points to compare across the two methods—definite on both datasets, and the basis of the proposed approach. The results showed that the ICM method provided values closer to the theoretical value of 2.0, but with noticeably greater standard deviation.

Visual inspection revealed that the peak approximation method initially selected was not precise enough, so the next step was to find a more accurate one. We chose to fit a normal distribution curve on the population histogram and use the expected value of this ideal distribution as the next step. As can be seen in Figure 12 and Figure 13, this resulted in a lower standard deviation (though still double that of the reference method) and a mean value closer to the theoretical expected value (Table 3).

The next stage was to compare the ICM results to the FCM results at the sample level, in an attempt to create a single transfer function mapping the proposed method to the reference.

Nonlinear methods were omitted for this stage of the experiment, due to the linear nature of the natural connection between the peak locations, which was also the reference and basis of the proposed approach. Using a nonlinear transformation on the data could change the relative relation of the measured 2N and 4N peaks, thus providing a better fit to the reference FCM data, but also possibly covering possibly important differences between the two data acquisition approaches. Moreover, the measurement appliance—the digital microscope—was shown to have a linear response to the change in exposure time on plastic beads [13]; we presumed the same behavior in the case of these nuclei. The basic idea was to find a scaling value that established the best mapping of the peaks. The two approaches measuring the same physical properties of the nuclei are visually represented on the graphical abstract, included as Figure 11.

To unite (or attempt to unite) the pairwise transformations into a single function for all the samples, two methods were implemented: the first was a naïve method (Figure 12) that simply uses the arithmetic mean of the scaling values, while the other was to use linear regression (Figure 14). The residuals of the regression fitting showed signs of non-normality. This prevented the application of some more robust techniques, and reduced the reliability of the linear model created. This usually occurs due to outliers or non-normality of the input data themselves. The sample size of these 17 samples is probably not sufficient for conclusive results in this regard.

On this topic, the naïve approach was informative. In Figure 12, the scaling component is visible. It shows that, although slightly less reliably, the ICM-fit method can produce similar results to the flow cytometry method. The mean value is closer to the theoretical 2.0 value, and the standard deviation is of the same magnitude compared to the FCM results, although almost double the value.

Both the RMSE and R-squared values (Table 4) could be significantly improved by removing the outlier samples that were probably used for determining the digitization parameters. The results after omitting data from samples #1, #12, and #18 (after visual inspection, all three of these samples were of considerably lower overall intensity) are visible in Table 5. These are significantly better measures, but in the case of such a low sample count, investigating the possible causes is important. The exposure and gain values defining the image intensities were fixed, and were the same for all of the samples, so the errors can have only a few sources—the sample, the preparation process, or the image segmentation. The experimental processes resulting in these settings were not thoroughly documented (partially because the glass slides had no identifier before digitization), so the experiment itself can be improved in that respect. In a real-life setting, where the main goal is the calibration itself, a higher number of samples and the use of a separate slide set for parameter setting would probably result in less uncertainty.

This study shows the differences between the two measurement methods, prioritizing the peak distance ratios. This is rooted in the initial goals—the evaluation of the image processing, the underlying segmentation, and the biological nature of the samples of the study. It answers the initial question—that the populations of the healthy samples’ 2C and 4C peaks are as present and detectable in the case of the ICM approach as they are with the FCM approach. Another way of describing the differences would have been to prioritize the 2C peak location and the measurement of the differences in that way. The input data being the same, the numbers, although with different values, would have the same meaning.

Dye bleaching is a phenomenon that causes fluorescent stains to fade with time and/or excitation. One aspect of sample digitization is the ability to circumvent this by storing a pristine state that can be re-evaluated without loss of quality. Digital slide scanners themselves are also gradually getting better at causing less and less bleaching during digitization, but the sample is often not lit in a homogeneous way, causing the dye in certain areas to bleach in a heterogeneous way. It would complement this study to take measurements for investigating this phenomenon, and even to formulate a compensation method. 

Other conditions of the sample affect the segmentation results (e.g., degrading cells are less intensive; their proportion in the sample is a marker of sample quality). Usual processes of sample acquisition, storage, and handling are recommended before similar measurements. A conference article [10] also explored the sample concentration’s effect on the segmentation accuracy.

## 5. Conclusions

The basic idea of using two-dimensional images instead of analyzing the passage of objects in front of a simple detector was shown to give similar information about the populations’ DNA contents.

The basic idea of using healthy samples in a lab to calibrate an image-based cytometry system is possible. The ICM method generated results regarding the measurement of the cell populations’ relative DNA content that were comparable to those of the FCM method.

Lab desk space is a fixed cost; the ability to consolidate tools can reduce that cost (not to mention the maintenance of multiple machines and operating personnel). In the case of diagnostic labs that are smaller in area but willing to expand in the direction of digital pathology, or those with a greater imaging workload, researchers might consider using the ICM approach—even as a replacement technology for FCM. In the former case, the introduction of ICM ploidy can be simpler, because the preparation process needs only to be extended with sample placement on glass slides and coverslipping. In the case of the latter, a glass-slide-only workflow can be implemented.

The inherent benefit of being able to store specimens for indefinitely longer periods is another important facet to consider; either glass slides or digital images can be of great importance as a database for later research projects.

When the continuous (or even automatic) calibration of such a system comes into view, fitting a linear model to create a function transforming one method of data measurement to the other based on the samples in this study does not seem possible. In Figure 15, samples #1, #12, and #17 are good examples, showing the difficulty in fitting a model—the same biological sample was represented as low-intensity by one system and high-intensity by the other (relative to the set population). The same sample’s histograms’ relative positions on the *x*-axis (i.e., the intensity, the DNA content) result in the necessity of quite different per-sample transformation functions, to which we failed to fit a linear model. Further investigation would be needed to confirm this system behavior, since neither method gave more consistent measurements than the other regarding this measure.

The sample count of 17 is also too low; a study aiming at direct calibration needs to be designed, and should include more samples to obtain more reliable statistical results.

## Figures and Tables

**Table 1:** PCA coefficient matrix of one of the samples.

Parameter	PC 1	PC 2	PC 3
Area	0.0002	−0.0036	**1.0000**
DNA Content	**0.9985**	−0.0547	−0.0004
Granularity	0.0547	**0.9985**	0.0036

**Table 2:** Median match counts for all samples.

Parameter	Median Match Ratio
DMG	100.00%
Prewitt	100.00%
Roberts	94.44%
Sobel	5.56%
Canny	16.67%

**Table 3:** Peak ratio properties of all of the samples combined.

Population	Mean	SD
FCM Histogram Peaks	1.8868	0.022131
ICM Histogram Peaks	1.9101	0.101460
ICM-Fit Peaks	1.9348	0.039905

**Table 4:** Linear regression results (values rounded to 4 decimal places).

Method	Scale (10^−4^)	Intercept	RMSE	R-Squared
ICM	4.8627	324.7997	135.0	0.284
ICM-fit	4.9973	318.3169	133.0	0.303

**Table 5:** Linear regression results after manual, visual-inspection-based outlier exclusion (values rounded to 4 decimal places).

Method	Scale (10^−3^)	Intercept	RMSE	R-Squared
ICM	1.0286	70.6179	94.0	0.832
ICM-fit	1.0281	67.6189	89.8	0.846

## Data Availability

The data used in this study can be made available upon reasonable request.
